# Recognition and Repetition Counting for Complex Physical Exercises with Deep Learning

**DOI:** 10.3390/s19030714

**Published:** 2019-02-10

**Authors:** Andrea Soro, Gino Brunner, Simon Tanner, Roger Wattenhofer

**Affiliations:** ETH Zurich, Department of Information Technology and Electrical Engineering, 8092 Zurich, Switzerland; soroa@ethz.ch (A.S.); wattenhofer@ethz.ch (R.W.)

**Keywords:** human activity recognition, har, smartwatch, imu, deep learning, repetition counting, exercise classification, sports analysis

## Abstract

Activity recognition using off-the-shelf smartwatches is an important problem in human activity recognition. In this paper, we present an end-to-end deep learning approach, able to provide probability distributions over activities from raw sensor data. We apply our methods to 10 complex full-body exercises typical in CrossFit, and achieve a classification accuracy of 99.96%. We additionally show that the same neural network used for exercise recognition can also be used in repetition counting. To the best of our knowledge, our approach to repetition counting is novel and performs well, counting correctly within an error of ±1 repetitions in 91% of the performed sets.

## 1. Introduction

Physical exercise is an important part of many people’s every-day life. For example, on any given day, around 20% of the U.S. population engaged in physical exercise in 2015 [1]. There are numerous studies that have found benefits of physical activity on mental and physical health [2]. While it is intuitively obvious to most people that physical exercise is beneficial, actually following a regular workout regimen can be difficult [3]. Having a community can help with motivation, but not everyone can/wants to join a club or participate in exercise classes. Luckily, there are now many wearable devices, such as fitness trackers and smartwatches, that include workout tracking and coaching features. Some of these devices even have entire ecosystems with the goal of increasing the motivation to work out (e.g., through online communities and challenges), and helping users to keep track of their progress. Most of the existing devices do well with tracking simple, long-lasting exercises, such as running, cycling and swimming. However, more effort is required to cover a wider range of sports, especially ones with fast-paced complex movements.

There has been a substantial range of work in the scientific community dealing with general strength training, where the recognition of different exercise types and the counting of repetitions is important. Automatically monitoring the performed exercises and number of repetitions is more convenient than having to manually keep track. Also, through automatic exercise tracking, large amounts of data are aggregated that can then be used to compute statistics to quantify progress over time. In other cases, one might want to follow a strict exercise plan that specifies the type of exercises and the number of repetitions to be performed. This is especially the case for CrossFit, where the so-called workouts of the day (WOD) specify exactly which exercises are to be performed, either for a fixed number of repetitions, or for as many repetitions as possible within a given time frame. Exercise recognition and repetition counting can help athletes to follow the workout schedule and automatically generate statistics about their progress. In this work, we therefore focus on 10 complex full-body exercises, as they typically appear in the sport of CrossFit. We present a deep learning approach for exercise recognition and repetition counting, based on convolutional neural networks (CNNs). In order to train and evaluate our models, we collect inertial data with two smartwatches, one worn on the wrist and one on the ankle. We collect data from a total of 54 participants of varying levels of athleticism and proficiency, yielding 230 min of data containing 5461 repetitions.

One major challenge with activity recognition is the collection of large amounts of accurately labelled data. This is often only possible with considerable human effort, including direct supervision during data collection, as well as analyzing video recordings to annotate the data. Therefore, we design a data collection scheme that allows collection of high quality data with minimal supervision. In particular, during this constrained workout, the smartwatch indicates the start of a new repetition through a vibration signal. Thus, we automatically acquire labels for the workout type, as well as the beginnings of repetitions. Such a constrained data collection approach decreases the difficulty of data acquisition and could, therefore, enable large scale crowd-sourced data collection. For example, fitness apps could include constrained workouts, thereby gathering an unprecedented amount of labelled data from millions of users and improve their machine learning models. Furthermore, such apps could also let users execute a few repetitions of exercises, which are then used to fine-tune the basic models. This form of personalization is a type of transfer learning, which, in itself, is an active area of research.

Our exercise recognition model achieves 99.96% accuracy. We further present a novel approach for repetition counting, where we use a neural network to detect the beginning of exercise repetitions. Our approach is straightforward and does not require any feature engineering, and we use the same neural network architecture for recognition and counting. For repetition counting, we achieve an error of ±1 for 91% of all sets. We also evaluate the importance of individual sensors and smartwatch locations and show that data from a single smartwatch, worn on the wrist, is sufficient to achieve a high accuracy for most exercises. However, we find that certain exercises clearly benefit from the second smartwatch. Wearing a smartwatch on the ankle might seem impractical at first, however, in a real-world setting, one could use lightweight inertial sensors like the Moov [4]. In order to stimulate future research and ensure the reproducibility of our results, we make our data and code publicly available (Data and Code: https://goo.gl/28w4FF).

## 2. Related Work

Human activity recognition (HAR) is an important sub-field of human-computer interaction (HCI), with a rich body of work. HAR covers a broad range of tasks, including distinguishing activity from non-activity [5,6], activity classification [7] and repetition counting [5,6,8,9,10]. These tasks are interesting by themselves from a research perspective, but also have a wide range of potential real world applications, especially in the fields of healthcare and personal fitness. This is exemplified by the increasing number of commercially available wearable devices that include activity tracking and coaching functionalities. For example, the Apple Watch [11] distinguishes between moving, exercising, and standing, and there is also support for exercise tracking, especially through third-party apps. Google’s Wear OS [12] also supports activity tracking natively and through third-party apps. Moov [4] is a dedicated fitness tracking system that consists of separate lightweight motion and heart rate trackers. One can also combine multiple motion sensors, e.g., wearing one on each wrist for boxing. HAR can be based upon many different modalities, such as video [13,14], sound [15], electromagnetic waves [16,17], and, probably most prevalent nowadays, body-worn inertial sensors [18,19], which we employ in this work.

Until recently, most work on HAR has used classical supervised machine learning methods to perform the tasks of segmentation and classification. The most widely used methods include decision trees, random forests, naive Bayes classifiers, hidden Markov models, and support vector machines. However, with the increased availability of data through off-the-shelf devices such as smartphones, smartwatches, and fitness trackers, as well a general increase in computing power, more work has focused on using deep learning methods [20]. In general, deep learning works exceptionally well for HAR and, in contrast to classical machine learning methods, requires little feature engineering. Convolutional Neural Networks (CNNs), variants of Recurrent Neural Networks (RNNs), and combinations of both methods have been shown to work very well on a range of HAR benchmark tasks [21,22,23,24]. In this work, we use a simple, yet effective, CNN architecture. One disadvantage of deep learning methods is the high computational cost, which makes them challenging to use in an online scenario on low power devices, such as smartwatches. Therefore, there has been considerable effort to design efficient deep learning methods without sacrificing too much performance [25,26,27]. We are interested in the best possible performance and thus conduct all the computation offline on GPUs. We leave the adaptation of our methods for efficient online computation as future work. For a detailed overview of deep learning methods for HAR, see [20]. In the following, we are mostly going to focus on HAR studies related to physical exercise.

Shoaib et al. [7] classified 13 activities from daily life using smartphone inertial measurement units (IMUs). Similar to us, they placed smartphones in two different locations (forearm, pocket) and found that using both devices improved recognition performance. O’reilly et al. [28] used a combination of 5 IMUs attached to the lower body to classify five different lower body exercises. They achieved 99% accuracy using a Random Forest. We considered twice as many exercises and achieved even higher accuracies, using only two IMUs. Also, our exercises included more complex compound movements, such as burpees and wall balls. Um et al. [29] used CNNs to classify 50 gym exercises and achieved 92.1% accuracy. The data they used were collected using the special-purpose PUSH [30] forearm-worn sensor that included an accelerometer and gyroscope. They used a large data set, provided by PUSH Inc., while we collected our own data and used off-the-shelf general purpose hardware. Ebert et al. [31] classified eight different body weight exercises, using an accelerometer on each arm plus a smartphone mounted to the chest. They used a naive Bayes classifier and achieved around 95% accuracy. In contrast, we considered more exercises, some of which were arguably more complex (burpees, wall balls), used fewer sensor devices, and achieved higher accuracies. Burns et al. [32] evaluated multiple machine learning algorithms to distinguish between shoulder exercises, based on inertial signals. They found that a Convolutional RNN (CRNN) outperformed classical approaches, and similar to us, they only used one off-the-shelf smartwatch. We additionally evaluated the benefit of using a second smartwatch attached to the ankle for detecting whole-body movements.

In the following, we will discuss a range of HAR approaches for sports and exercise recognition which are most closely related to our work, as they perform not only exercise recognition, but also repetition counting. Chang et al. [8] collected data using hand- and waist-mounted accelerometers and achieved 90% recognition accuracy on 9 typical strength training exercises. They also performed repetition counting, where they achieved a relative error rate of 5%. The authors used classical techniques such as naive Bayes, hidden Markov models, and peak detection, whereas we exclusively employed neural networks for recognition as well as counting. Seeger et al. [9] classified 16 typical daily, cardio, and strength training activities with 92% accuracy. They used data from three accelerometers (hand, arm, leg) and modeled each sensor axis as a Gaussian distribution (mean and variance) that was then directly used for recognition. They performed repetition counting using autocorrelation for slower exercises, and a variant of peak detection for faster exercises. Muehlbauer et al. [5] classified 10 different upper body exercises, with an accuracy of 93.6%, using a smartphone placed in an arm holster. They evaluated multiple classical algorithms for recognition and found k-nearest neighbours (kNN) to work best. Similar to [9], they also used auto-correlation for repetition counting. Morris et al. [6] built and improved on the aforementioned works. They used an arm-mounted inertial measurement unit and achieved up to 99% recognition accuracy across 14 gym exercises (plus walking and running). They also used an auto-correlation based peak detection algorithm for repetition counting and achieved impressive results, with an accuracy of ±1 in 93% of sets. Shen et al. [10] introduced a workout tracking application called MiLift. MiLift also performed segmentation, recognition, and repetition counting using similar approaches to the previously mentioned works.

In contrast to the aforementioned works, we used off-the-shelf smartwatches instead of smartphones or dedicated IMU units. We compared using a single smartwatch worn on the wrist to using an additional smartwatch worn on the ankle. With the exception of [6], most previous works mainly focused on single-joint exercises (e.g., bicep curl) or other, mostly stationary, exercises (e.g., bench press), while we chose ten full body exercises that included complex movements, such as burpees which consist of multiple “sub-exercises”. The exercises we chose vary greatly in execution time and form across athletes, making this a challenging task. The most crucial difference to previous work is that we employed an end-to-end deep learning pipeline for recognition as well as counting. To the best of our knowledge, we present a novel approach to repetition counting. One advantage of our approach is that it does not require any feature engineering at any stage in the pipeline. For recognition, we achieve a very high accuracy of 99.96% when using both smartwatches, and 98.91% when only using the wrist mounted watch. For counting, we are within ±1 for 91% of sets. Note that direct comparison with related work is difficult, since there are many differing factors such as sensor hardware, sensor placement, exercises, amount of data, nature of annotations, and so on. Nevertheless, we believe that our results are strong and they can only improve with more data.

## 3. Data

Currently there exist no publicly available data sets of sensor recordings for CrossFit. Therefore, we designed different workouts and collected data from volunteers. The data was collected using two smartwatches, one worn on the wrist and one on the ankle. This sensor data was then used for training and testing of the different models.

### 3.1. Exercise Selection

**Figure 1:**
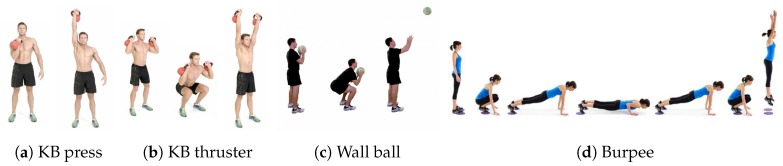
Execution of four typical CrossFit exercises. In our setting, the kettlebell (KB) thruster (**b**) is performed with only the right hand.Execution of four typical CrossFit exercises.

We picked ten of the most frequent movements performed in CrossFit, as listed in Table 1. As the reader might not be familiar with some of the more CrossFit-specific exercises, Figure 1 shows the execution of the kettlebell press (E9), kettlebell thruster (E10), wall ball (E8) and burpee (E3).

Several typical CrossFit exercises are Olympic lifts, and can only be performed safely with knowledge about their correct execution. To allow CrossFit beginners to participate in our study, exercises requiring a barbell were replaced by beginner-friendly unilateral versions using a kettlebell (exercises E4, E9, and E10 in Table 1). The kettlebell versions of these exercises are also widely used across all experience levels. They are less likely to cause injuries than the barbell versions, and are technically easier to execute. During the one-handed kettlebell exercises (E9 and E10), the kettlebell was held in the right hand, where the smartwatch was worn.

The exercises were chosen to have a large variety in execution time and body part engagement between different exercises. Additionally, some exercises with similar movements were included, to investigate how well they could be distinguished by the models. For example, in both the kettlebell thruster and wall ball, one first lowers into a squat and then straightens the arms when standing back up. Similarly, the end of the kettlebell thruster and kettlebell press are both essentially a shoulder press. Furthermore, the burpee contains a push-up and a jump, similar to a box jump.

#### 3.1.1. Constrained Workout

The constrained workout was designed to give accurately labeled sensor data, including the type of exercise and the start of each repetition. This was achieved by indicating the start of the repetitions to the participant, using vibrations of the smartwatch (worn on the wrist). Note that this makes additional data labeling steps, such as taking a video of the workouts for subsequent refinement of the annotations, superfluous. This is advantageous as it reduced the data collection effort, but also made it easier to find willing participants, since many people do not like being recorded on video. Drawbacks might be a loss of generality since participants had to wait for the vibration signal before starting the next repetition. However, we tried to keep the negative effects of this choice to a minimum by setting a personalized vibration interval for each participant and exercise. The vibration interval was set such as to reflect the natural execution speed of the exercises by the participant, as closely as possible.

For some exercises (e.g., E3, E8, E10) waiting for the next vibration interrupted the natural flow of the exercise. In these exercises, the end of one repetition normally tended to overlap with the beginning of the next. This is especially the case in CrossFit, where exercises are usually performed as fast as possible and the form is less strict than in the case of normal strength training. For example, when push-ups are executed strictly, there is a short but distinctive pause at the top where both arms are fully extended. In CrossFit, athletes tend to stop just short of full extension and fluidly drop back down for the next repetition. This holds true for all exercises, albeit to varying degrees. Therefore, this could affect the performance of the models when used on data recorded without a fixed execution speed. However, there would be no issue when applying our models to strength training done in sets and repetitions, and hence with “normal” speed, instead of for time.

The participants were asked to perform one set of 15 repetitions of each exercise. However, they were free to stop earlier and were encouraged to do extra repetitions in the case that they were not over-fatigued. This allowed us to collect as much data as possible of the exercises performed with good form. After each set, the participants were free to rest before moving on to the next exercise.

#### 3.1.2. Unconstrained Workout

In the unconstrained setting, we let the participants freely execute the exercises with a focus on fast execution, as is typical in CrossFit. In this case, the smartwatch did not vibrate to indicate the start of a new repetition. The data collected in the unconstrained setting was only used for testing, and not for training.

We define two workout types. The first type is similar to the constrained case. Here the participants performed all of the 10 exercises with 10 repetitions each. This setting allows us to test how our models perform on data that is not paced by the vibrations of the wrist watch. In particular, the clear beginning and ending of single repetitions are not present anymore and, instead, consecutive repetitions have smooth transitions. This especially poses a challenge for repetition counting.

The second unconstrained workout type follows a 1-2-3 scheme, where in the first round all 10 exercises were performed for one repetition, in the second round for two repetitions, and in the third round for three repetitions. This scenario enables us to evaluate how many consecutive repetitions are needed for robust classification. In general, the shorter a single exercise was performed for, the harder it is to recognize.

#### 3.1.3. Null Class

The unconstrained workouts were done in a circuit fashion, where participants moved freely from one exercise to the next. Additionally, some of the exercises required kettlebells and medicine balls, which the participants had to pick up. Therefore, it was crucial to include non-exercise, or null class, training data to correctly identify periods where no exercise was performed. In order to collect null class data, we had participants sit, stand, and walk around for 90 to 120 s.

### 3.2. Data Collection App

**Figure 2:**
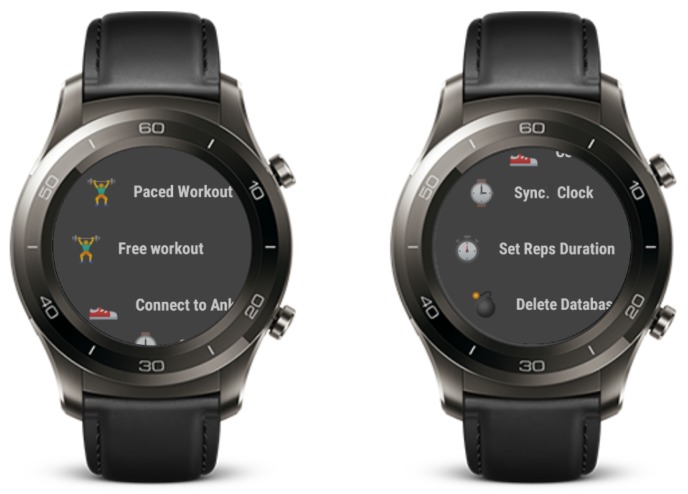
Main menu screen. Participants choose the workout type and set the vibration interval duration.

The sensor data was collected with an app that ran on two smartwatches in communication with each other, over Bluetooth low-energy (BLE). The main menu of the app can be seen in Figure 2. This screen was displayed when the app started and allowed choice of one of the workouts, connection of the two smartwatches over Bluetooth, and synchronization of the clocks. Additionally, the time interval of the vibrations that signalled the start of a repetition could be set.

Once the Bluetooth connection between the two devices was established, the wrist watch sent commands to the ankle watch and no further user interaction on the ankle watch was needed. The screen on the ankle watch was then just used to check that everything was working correctly during the workout, and touch interactions were disabled.

To start the recording of the sensor data simultaneously on both watches, they had to be synchronized in time. For simplicity, we solved this with the NTP library TrueTime [33]. Both watches were connected to a WiFi network and obtained the correct time from the NTP servers.

**Figure 3:**
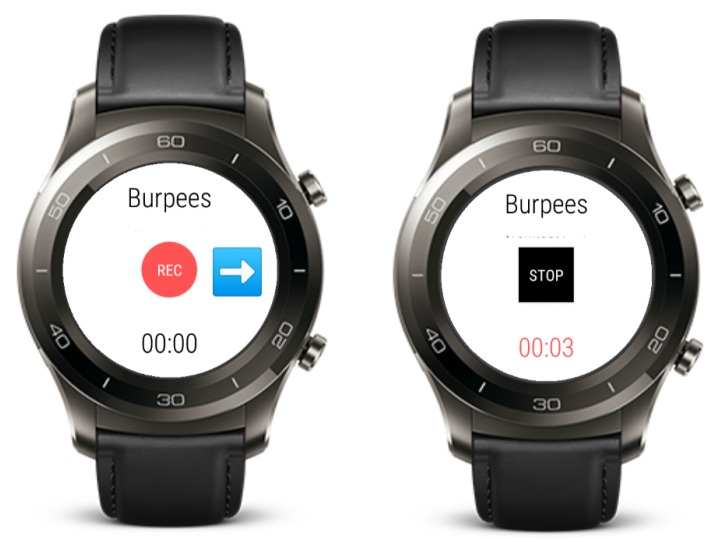
Workout screen. The left image shows the state before starting the recording. The right image shows the state during the exercise.

The workout screen, shown in Figure 3, was displayed during the data collection. This screen showed the current exercise and a button for starting and stopping the recording of the sensor data. The watch started recording after a 5 s countdown, to allow the participant to get into position. After stopping an exercise, the participant input how many repetitions they had performed. The app ran on two Huawei Watch 2 smartwatches with Android Wear 2.9. The two watches were always worn in the same orientation on the right ankle and the right wrist and were tightened enough to avoid rotation.

**Figure A1:**
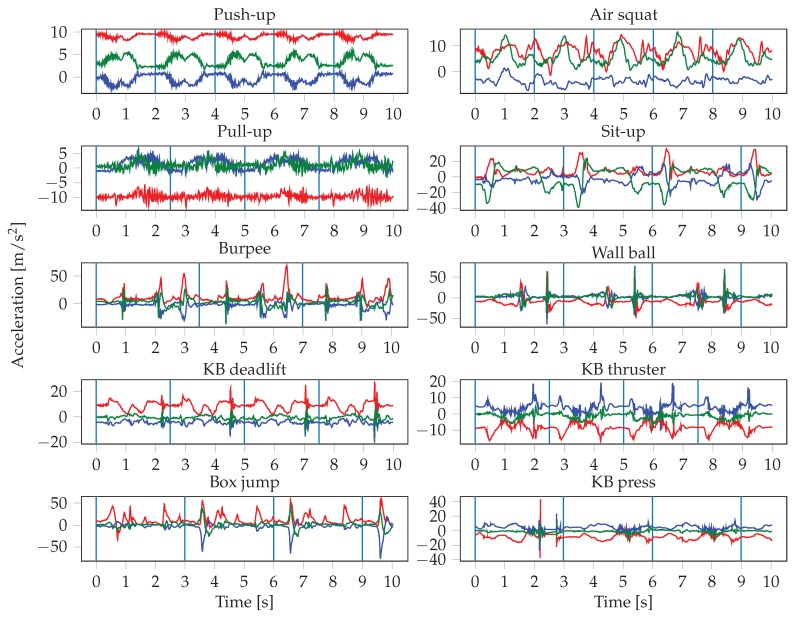
Accelerometer raw readings for 10 s for all exercises.

**Figure A2:**
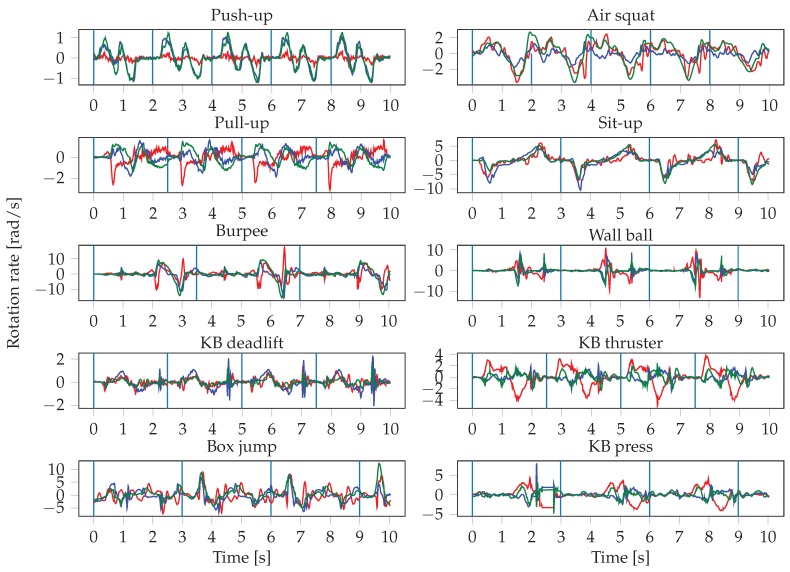
Gyroscope raw readings for 10 s for all exercises.

**Figure A3:**
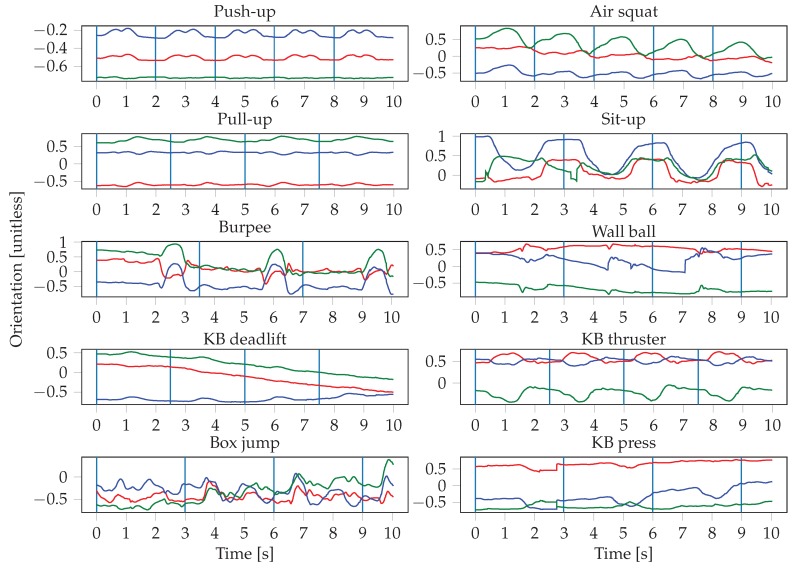
Orientation raw readings for 10 s, for all exercises.

The data of the accelerometer, the gyroscope, and the rotation vector sensor were recorded during all exercises with a frequency of approximately 100 Hz. The rotation vector sensor is a software sensor that gives the orientation of the watch. The other two are hardware sensors that measure raw data about the linear and rotational forces that are applied to the watch. We directly used the raw data provided by these sensors without applying any post-processing, such as low-pass filtering or drift compensation. An overview of all the motion sensors on the Android platform and how to access them can be found here [34]. The accelerometer measures the acceleration (including gravity) along the *x*, *y*, and *z* axes, in m/s^2^. The axes are defined in relation to the device, where the *z* axis is perpendicular to the screen (see [35]). Figure A1 shows an example of raw acceleration data. The gyroscope measures the rotational velocity, in rad/s, around the *x*, *y*, and *z* axes, where the coordinate system is the same as for the accelerometer. Figure A2 shows an example of raw gyroscope data. Finally, the rotation vector sensor measures the orientation of the watch (azimuth, pitch, roll) in the earth’s frame of reference. The orientation angles are computed by combining data from the geomagnetic field sensor (compass) and the accelerometer. Figure A3 shows an example of orientation sensor data.

During the data collection process, the data streams from the three sensors were annotated with the performed exercise type, and repetition starts were indicated using the vibrations of the wrist watch.

### 3.3. Collected Data Summary

A total of 61 people volunteered to take part in the data collection. The data of 7 participants had to be excluded, because of technical problems with the smartwatches—sometimes, the ankle watch stopped recording data or the watches did not stay synchronized. Out of the remaining 54 people, 50 participated in the constrained workout. The unconstrained workout was performed by five people, of which four also participated in the constrained workout. The null class data was collected from seven individuals, of which four also took part in the constrained workout. The participants are between their early 20’s and early 40’s. Of the participants, 43 were men and 11 were women. The CrossFit experience level of the participants could be divided into three levels: Beginner, intermediate, and advanced. Beginners are participants that have had less than 6 months of practice, intermediate ranges from 6 up to 24 months of weekly or semi-regular practice, and advanced practitioners have performed the sport for at least 2 years with high frequency. Of the people that participated in the constrained workout, 16 were beginners, 22 intermediate, and 12 advanced. Six of the advanced participants were certified CrossFit coaches. In the unconstrained workout, three participants were intermediate and two were advanced. Having data from participants with such different experience levels helps to obtain a model that adapts well to many users. Some of the beginners in the study have never before performed some of the movements. Table 2 gives an overview of the collected data.

## 4. Methods

**Figure 4:**
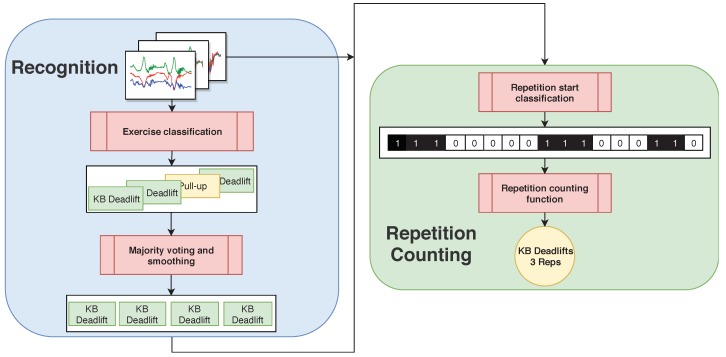
System pipeline. For exercise recognition, raw sensor data is split up into windows and fed to a CNN that performs exercise classification. For repetition counting, the windowed raw sensor data is fed into the repetition counting CNN, which corresponds to the exercise that was previously detected by the recognition CNN.

Figure 4 shows a high-level overview of our pipeline. Windows of raw sensor data are fed into an exercise classification neural network that predicts the exercise type for each window. We use overlapping input windows, majority voting, and subsequent smoothing. For repetition counting, we use separate neural networks, one for each exercise type. Thus, in order to choose the right neural network for counting, correct exercise recognition is crucial. The repetition counting neural network outputs a series of 1’s and 0’s, where 1’s designate the start of a repetition. We, then, apply smoothing to this binary series and count the number of 1-sequences, which gives the final repetition count. In the following, we will describe the individual steps of the pipeline in more detail.

### 4.1. Neural Network Architecture

**Figure 5:**
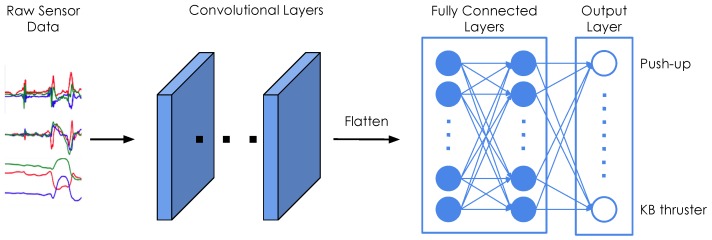
Neural network architecture for recognition and counting. Windows of raw sensor data from up to six sensors are passed through several convolutional layers, before a fully connected layer with a softmax activation outputs the final predictions.

We use the same base architecture for both exercise recognition and repetition counting, as shown in Figure 5. All our classifiers consist of a series of 2D convolutions and two fully connected layers. We fine-tune our models through hyper-parameter grid searches. Table 3 summarizes the hyper-parameter search for the recognition model, and indicates the best values that were found. We note that the results are, overall, robust to these hyper-parameter choices and we thus refrained from further tuning. A complete list of the architecture parameters can be found in Table A1 in Appendix A. We further tuned the models for each exercise type individually, according to the values in Table 4. The final hyper-parameters of the repetition counting models for each of the exercises can be found in Table A2 in Appendix B.

The sensor readings were obtained at irregular intervals, therefore the signals are first interpolated and resampled. The resampling is performed with a frequency of 100 Hz, which represents the average frequency of the watch sensors. The input to the neural networks consists of the resampled sensor data from multiple sensors, where each sensor has three axes. Since we record the signals of the accelerometer, gyroscope, and orientation sensor of two smartwatches, this results in a total of 18 inputs. One input window $X_{i}$ is a matrix of dimensions T×100×3×S, where *S* denotes the number of sensors that were used, and *T* is the length of the input window in seconds. This means that we stack the sensors on top of each other in a single channel. In the first convolutional layer, we therefore use a stride of 3 along the vertical axis, in order not to arbitrarily mix together neighbouring axes of different sensors. In our experiments, we evaluate the importance of sensor types and locations, thus *S* ranges from 1 (when only using one sensor of one smartwatch) to 6 (when using all sensors from both smartwatches). The choice of the input window length *W*, defined as W=T×100, was crucial, especially when dealing with activities of different lengths. In general HAR, one might want to recognize long-running activities such as reading, but also detect short actions, such as taking a sip of water, which might require operating at different time scales (i.e., using multiple different window lengths). In our case, the differences in duration between the shortest (push-up) and longest exercise (burpee) are relatively small, which should allow for use of a single window size. We evaluated different choices for *W*, ranging from 100 to 1000 sensor samples (1 to 10 s).

Our neural network models are implemented with Keras [36] using the Tensorflow [37] backend. We use a batch size of 30, and train for a maximum of 100 epochs. For each batch, the input windows $X_{i}$ are sampled independently and uniformly at random from the training data set. We use early stopping, where training is finished early if the test error does not decrease for a number of epochs. For training, we use stochastic gradient descent with a learning rate of 0.0001 to minimize the cross-entropy loss. We experimented with different optimizers, but did not find a significant difference in performance.

To evaluate the performance of our models we either use 5-fold cross validation with a 80/20 split for each fold, or leave-one-subject-out (LOSO) training. In both cases, we make sure that all exercises of each participant either end up in the training or the test set. In the case of 5-fold cross validation, only 5 models are trained, whereas, for LOSO, the number of trained models equals the number of participants, which was 51 in our case. Therefore, we only use LOSO for the repetition counting, where each model is effectively trained on only 1/10 of the training data, and we thus use LOSO to get a more robust estimate of the model performance and maximize the amount of available training data.

Note that we do not use a separate validation set for tuning the hyper-parameters, as is standard in machine learning for most supervised learning tasks. One of the biggest challenges we are facing is that, even though our data set contains a relatively large amount of data (13,800 s, 5461 repetition), the data of any given participant is highly correlated. Therefore, data from one participant must entirely be either in the train or in the test set. This is different to most supervised learning settings, where the train and test sets are uniformly sampled from the entire data set. Assume we were to use a standard 80/20 train/test split (and then split the train set again into train/validation). This would mean assigning all the data from 40 participants to the train set, and the data from the remaining 10 participants to the test set. If we, now, train a single model and test it on this particular test set, the resulting performance estimate would be highly dependent on which 10 individuals were assigned to the test set. For example, let us assume we have two groups of participants that are quite different from one another (e.g., 40 beginners and 10 advanced athletes). If, by chance, the 10 advanced participants end up in the test set, the performance on the test set will likely be low, because the model never saw advanced athletes during training. This is, of course, a simplified example, but it shows that, in order to get a robust estimate of performance, it is necessary to average over many possible choices of train/test splits. This is why we perform 5-fold cross validation, and even LOSO training for the case of repetition counting, in order to get a performance estimate that is independent of the particular choice for the train/test split. If we, now, wanted to use a validation set for hyper-parameter tuning in order to avoid overfitting the hyper-parameters on the test set, we would additionally need to split every train set into separate train and validation sets. The same reasoning as above applies (i.e., we need to consider many such train/validation splits in order to get robust performance estimates for each hyper-parameter setting). Therefore, we would need to perform nested k-fold cross-validation, which, for a choice of k=5, would result in having to train 25 models for a single hyper-parameter setting. Clearly, this becomes prohibitive even for small search grids, especially since we would need to perform this process a total of 11 times (once for the recognition model, 10 times for the repetition counting models).

In order to reduce the risk of overfitting hyper-parameters on the test set, we only perform minimal hyper parameter tuning. We start with a sensible architecture, based on which we perform a limited grid search. We want to emphasize that many hyper-parameter combinations perform almost equally well across the data from all participants. Therefore, we conclude that it is unlikely that we significantly over-estimate the generalization performance.

### 4.2. Exercise Recognition

For exercise recognition, each input window $X_{i}$ is labeled with one of the 10 exercises, and the neural network tries to predict the correct label. For the unconstrained workout data, there is an additional label for the null class. During test time, we overlap consecutive windows by a factor of γ, with 0 ≤ γ < 1. If γ is greater than zero, we use majority voting to get a single prediction for each point in time. Intuitively, the larger the overlap, the more accurate and robust the final predictions should be. The output of the neural network is a sequence of predicted exercise labels $\left(\ldots ,E_{i}^{t-1},E_{j}^{t},E_{k}^{t+1},\ldots \right)$ with i,j,k∈{0,…,9}, and where $E_{j}^{t}$ denotes that exercise $E_{j}$ is predicted at time *t*. In order to further improve the final results, we smooth the predicted sequence. We, first, look up the minimum repetition duration $T_{i}^{min}$ for each exercise in our data set, which is equal to the shortest vibration-interval for that exercise used during data collection. We, then, go through the output of the neural network and find all sequences of the form $\left(\ldots ,E_{j}^{t-1},E_{k}^{t},E_{j}^{t+1},\ldots \right)$, where j≠k. For each of these sequences, we then replace $E_{k}$ with $E_{j}$ if the duration of $E_{k}$ is shorter than $T_{k}^{min}$.

### 4.3. Repetition Counting

Since we have used a vibration signal during data collection that signals the start of each repetition, we obtain labels for the beginnings of repetitions. We then train a neural network to recognize whether an input window contains a repetition start. As explained in Section 4.1, we use the same basic architecture as for exercise recognition. In contrast to exercise recognition, where we use a single window size, we use a separate window size for each exercise. The length of the window for exercise $E_{i}$ is set to the shortest vibration-interval $T_{i}^{min}$. This ensures that one window will never fully contain two repetition beginnings. We, then, define the start of a repetition as the first $T_{i}^{min}/2$ samples after the vibration signal. We label the input windows $X_{i}$ with 1 if and only if the entire start of the repetition is contained in $X_{i}$, and with 0 otherwise. Thus, the problem of counting repetitions is reduced to a binary classification problem. The output of the repetition counting network is a binary sequence (e.g., as in Figure 4). If the network output was perfect, counting the number of 1-sequences would yield the repetition count. However, as this is not always the case and many errors can be easily recognized by inspection, we further smooth the binary sequence to improve the results. Thus, we first determine the repetition mode $M_{1}$ by finding the most frequent 1-sequence length. This gives us a good estimate of how many consecutive 1’s constitute an actual repetition. We, then, find all 1-sequences that are shorter than $M_{1}/2$ and mark them as candidates for removal. All 1-sequences of length at least $M_{1}/2$ are considered as confirmed repetition starts. We, then, determine the repetition mode $M_{0}$ of the 0-sequences in the same manner. For each of the candidates, we check if they are at realistic distances from a confirmed repetition start and, if not, we set that candidate to 0. We define a candidate as realistic if there is no confirmed repetition starting $M_{0}/2$ before and after it. Finally, we count the remaining 1-sequences, which yields the repetition count.

## 5. Experimental Results

In this section, we present the results of exercise classification and repetition counting on the constrained and unconstrained workouts.

### 5.1. Recognition

#### 5.1.1. Constrained Workout

We evaluate the recognition of the performed exercises using 5-fold cross validation and achieve a test accuracy of 99.96%, shown in Table 5. To evaluate which sensors are most useful for recognizing the exercises, Table 5 further shows the performance of different sensor combinations. This also shows us the benefit of the second smartwatch on the ankle. All the models are evaluated using 5-fold cross validation. The best performance is achieved using the data from all sensors combined. Using only the sensors of the wrist watch, the performance drops slightly, whereas using only the ankle watch results in significantly lower accuracy. Therefore, only using a single wrist-worn smartwatch, which is the most practical case for everyday use, it is possible to achieve very high accuracy. When it comes to sensor types, the most information is gained from the accelerometer. Adding the gyroscope improves accuracy significantly, as well. The orientation sensor generally does not help or hurt the performance.

**Figure 6:**
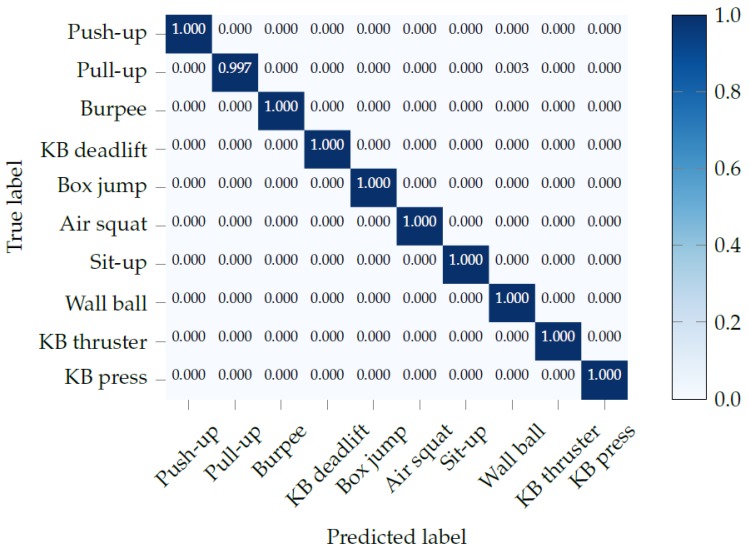
Confusion matrix of exercise recognition using all sensors from both smartwatches.

**Figure 7:**
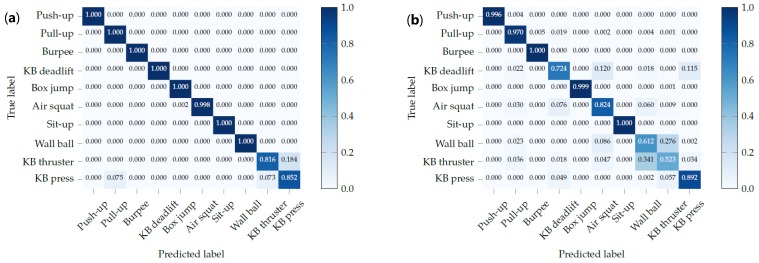
Comparison of recognition performance when only using the wrist or ankle watch respectively. (**a**) Confusion matrix of exercise recognition using only the sensors of the wrist watch. (**b**) Confusion matrix of exercise recognition using only the sensors of the ankle watch.

The confusion matrix, shown in Figure 6, shows that the recognition performance when using all sensors from both watches is nearly perfect. The additional information provided by the ankle-mounted watch can help distinguish between exercises with similar arm movements, such as kettlebell (KB) thrusters and KB presses. This can be seen in the confusion matrix, in Figure 7a, where those two exercises are confused. Adding the ankle watch completely solves this ambiguity. On the other hand, Figure 7b shows the confusion matrix when only using the ankle watch. Half of the exercises are recognized with an accuracy above 97%. As one might expect, the exercises where participants are standing with both feet solidly on the ground are confused here (KB deadlift, air squat, wall ball, KB thruster, and KB press).

While adding the sensor data from the ankle watch improves the overall results, it also causes the recognition accuracy for pull-ups to drop slightly. This could simply be due to the inherent variance between training runs of neural networks. Re-training the networks with a different random seed, or stopping the training process at a different time, would likely result in the optimization algorithm finding in a different local minimum, which in turn could produce slightly different results.

**Figure 8:**
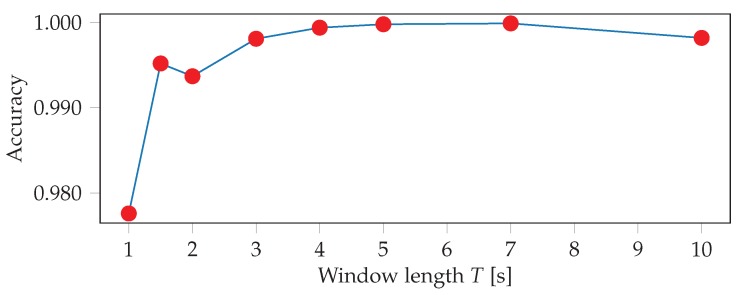
Five-fold cross validation test accuracies for various input window lengths *T*.

**Figure 9:**
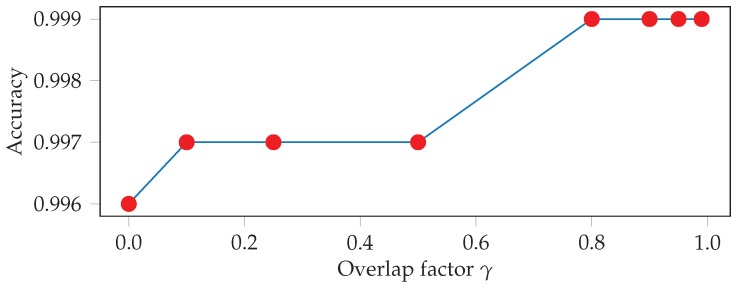
Five-fold cross validation test accuracies for various overlap factors γ.

Additionally, we investigate the effect of the input window length and overlap of the test windows on the detection accuracy. The best accuracy is achieved with a window length of 7 s, as is shown in Figure 8. This makes sense since during the constrained workout participants are performing the same exercise for long periods of time, similar to swimming or walking. However, we also want our models to perform well when exercises are only performed for short periods of time, such as in our 1-2-3 style unconstrained workout setting. We therefore select a final window length of 4s for all further exercise recognition experiments. Using overlapping windows and majority voting at test time also improves the results, which is shown in Figure 9. Overall, our model seems to be robust against these hyper parameter choices, as the differences between the worst and best performance are within less than 1%.

#### 5.1.2. Unconstrained Workout

We investigate how our method performs, when tested on two forms of unconstrained workouts. In the first workout, participants were asked to freely perform all 10 exercises in a row for 10 repetitions. We collected data from five participants, and used this data for testing only. We observe that our model generalizes well to the unconstrained setting, and is able to correctly classify almost all 10 exercises for all 5 participants, as can be seen in Table 8. Only for participant P4 did the model fail to correctly recognize the air squat exercise, and classified the movements as wall-balls instead. However, this is an understandable mistake, since wall-balls basically consist of an air squat with a slightly different arm motion in order to throw the ball. More training data could solve this problem. Note that, since we do not have the exact repetition start times, we cannot verify how accurate the predictions are in time. As ground truth, we only know the correct order of the exercises and the number of repetitions. However, together with the repetition counting, discussed later (see Section 5.2.2), we get a good estimate of how well our model generalizes to the unconstrained setting.

**Figure 10:**
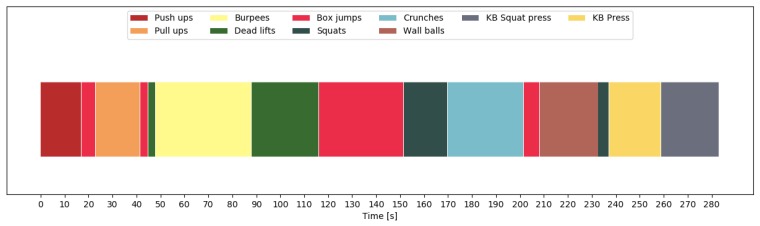
Chronological sequence of recognized exercises during free workout one for participant 1 (P1). The recognition is done without including the non-exercise class, thus causing the model to interpret transition as exercises.

**Figure 11:**
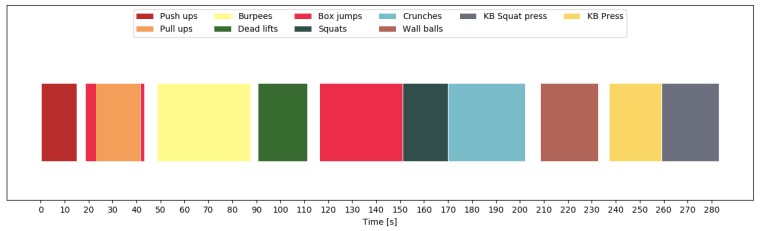
Chronological sequence of recognized exercises during free workout one for P1. The recognition is done including the non-exercise class, but without filtering out short-duration predictions. Most transitions are now correctly identified as such.

**Figure 12:**
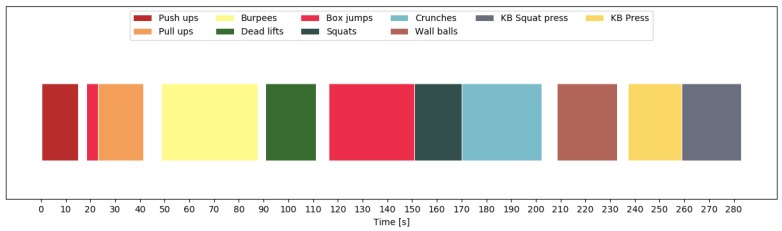
Chronological sequence of recognized exercises during free workout 1 for P1. The recognition is done including the non-exercise class and filtering out short-duration predictions. Most transitions are now correctly identified, and the short box jump after the pull-up is filtered out.

In order to show the importance of including null class data, we look at the predictions for one participant. Figure 10 shows the predictions of all exercises and transition periods, without having trained on null class data. Clearly, the exercises were recognized correctly without any discontinuities within the 10 repetitions. However, the transitions were sometimes recognized as different exercises, especially when transitioning between exercises that required moving around or changing equipment. For this participant, our model recognized a deadlift before the burpees, a box jump before the wall balls, and a squat before the kettlebell press. Figure 11 shows what happened when we included the null class. We now see a clear separation between exercises, and most of the aforementioned mistakes were avoided. Finally, Figure 12 shows the final predictions after smoothing out unrealistically short exercise sequences. This got rid of the short box jump after the pull-up sequence. The final mistake, that was still left, is the box jump before the pull-up. However, this mistake can be easily explained, since the participant jumped up to the pull-up bar, which was falsely recognized as a box jump. This error could potentially be avoided by including this kind of transition in the null data.

The second unconstrained workout type follows a 1-2-3 scheme. Table 6 shows how many of the 10 exercises were correctly recognized. Clearly, when the participants only performed a single repetition, recognition is more difficult, as every exercise was only performed for a short time period. Starting from two consecutive repetitions, our model already recognizes nearly all exercises correctly for all participants. As performing a single repetition of an exercise is a rather exotic case, even in the context of CrossFit, we conclude that our model generalizes well to realistic unconstrained workouts.

### 5.2. Repetition Counting

The repetition counting is evaluated using leave-one-subject-out (LOSO) training to maximize the amount of training data. We use various statistics to show the performance of the repetition counting: The mean absolute error (MAE), the mean relative error (MRE), and the percentages of exercises for which the number of counted repetitions is off by 0, 1, 2, or more than 2. The MAE is the average over all sets of one exercise of the absolute error between the predicted and the actual number of repetitions, and the MRE is the average of the relative error over the sets of one exercise.

#### 5.2.1. Constrained Workout

**Figure 13:**
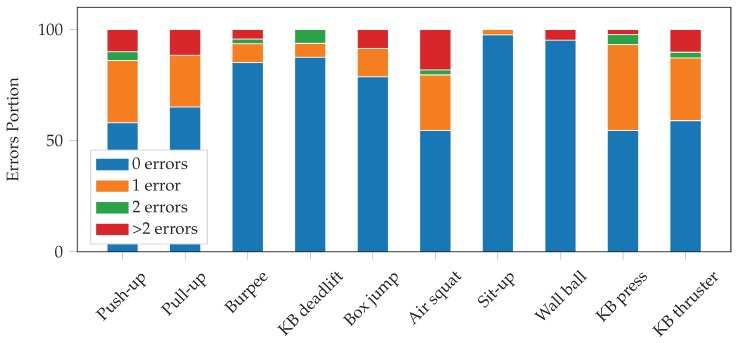
Number of errors of the repetition counting on constrained workout data for the different exercises. The exact values can be found in Table 7.

Table 7 shows the repetition counting results for the constrained workout. We achieved a mean absolute error (MAE) of 0.7 repetitions per set, and a mean relative error (MRE) of 6.1%. In 74% of the sets, the model perfectly predicts the number of repetitions performed. In 91% of the sets, it is off by at most 1 repetition. For 7% of the sets, the count is off by more than 2. We can see that the performance varies significantly between the different exercises. The repetition counting is very accurate for burpees, KB deadlifts, box jumps, sit-ups, and wall balls. For some exercises, such as push-ups, air squats, and kettlebell thrusters, however, the performance is worse. The length of the individual repetitions could have an influence on the performance. Many of the exercises with good performance had longer durations. However, KB deadlifts also perform well and had rather short repetitions. Figure 13 visualizes the number of errors for each exercise.

#### 5.2.2. Unconstrained Workout

We also test the repetition counting model on the unconstrained workout, where each participant performed 10 repetitions. The results are shown in Table 8. In most cases, the model is off by at most 2 repetitions. Again, the performance depends on the exercise. The repetition counting model performs slightly worse on the data than the unconstrained workout. The decrease in performance for wall balls and push-ups was to be expected. The waiti for the next repetition, signalled by vibrations in the constrained workout, interrupted the flow of these exercises. In the unconstrained workout, however, the end of one repetition flowed into the beginning of the next. Therefore, the model trained on the data from the constrained workout performs worse, when tested on data from the unconstrained workout.

The repetition counting performs similarly in the unconstrained 1-2-3 scheme. The repetition scheme should not affect the repetition counting, as long as the exercise is correctly recognized. This is confirmed by the results in Table 9. Note that the first participant followed a 1-3-5 scheme.

## 6. Conclusions and Future Work

In this paper, we present a deep learning method for exercise recognition and repetition counting for complex full-body movements. We reached 99.96% recognition accuracy on the constrained exercise data, and we showed that our model generalized to two different types of realistic unconstrained workouts. Our counting method was based on recognizing the beginning of exercises and we achieved ±1 error for 91% of sets. This approach is straightforward and does not require any feature engineering or post processing, and we showed that it works well with relatively small amounts of training data.

In the future, we plan to collect more data, especially for the unconstrained workouts, so we can improve the evaluation of the constrained models, and potentially incorporate it during the training. We found that repetition counting did not generalize well from the constrained to the unconstrained setting for certain exercises. This is especially true when exercises were executed in fast succession, such that the transitions between repetitions became smooth. In order to remedy this, we plan to annotate exercise repetition starts during unconstrained workouts by using video recordings.

Even though we plan to collect more unconstrained data, data collected in the constrained setting will still make up the majority of our data. Therefore, we plan to gain a better understanding of the impact of the constrained data collection on generalization performance. By doing so, we will be able to improve the data collection to mitigate negative effects. In order to take full advantage of the possibilities of the constrained data collection approach, we plan to publish an app to crowdsource the data collection.

In this paper, we built a pipeline to classify exercises and count repetitions in an offline manner. In order to make our system accessible to many people, one could implement it as an Android Wear OS application. This would bring numerous engineering challenges, in order to be able to run our neural network models on limited memory and computation resources. To reduce resource consumption, one could, for example, compress the neural networks or down-sample the sensor data. Reducing the overlap of consecutive input windows would reduce the computation cost, as well. Running neural networks on embedded platforms is an active area of research in itself. Alternatively, the computations could be performed in the cloud.

## Figures and Tables

**Table 1:** List of all exercises with respective code and equipment used.

Ex. Code	Exercise	Equipment
E1	Push-up	Body weight
E2	Pull-up	Pull-up bar
E3	Burpee	Body weight
E4	Kettlebell deadlift	Kettlebell
E5	Box jump	Box
E6	Air squat	Body weight
E7	Sit-up	Body weight
E8	Wall ball	Medicine ball
E9	Kettlebell press	Kettlebell
E10	Kettlebell thruster	Kettlebell

**Table 2:** Statistics of the collected data.

Exercise	Participants	Time [min]	Repetitions	Fraction of Time
Push-up	50	25.05	718	11%
Pull-up	43	13.63	300	6%
Burpee	47	30.97	551	13%
Kettlebell deadlift	48	28.43	689	12%
Box jump	47	19.2	546	8%
Air squat	44	22.27	642	10%
Sit-up	42	26.5	555	12%
Wall ball	42	23.42	529	10%
Kettlebell press	44	19.7	482	9%
Kettlebell thruster	39	20.88	449	9%
Total	-	230.05	5,461	100%

**Table 3:** Parameters of the grid search for the recognition model.

Parameter	Candidates	Best
Number of convolutional layers	1, 2, 3, 5	5
Filter convolutional layer 1	25, 50, 75, 100	100
Filter convolutional layer 2	25, 50, 75, 100	75
Filter convolutional layer 3	25, 50, 75, 100	25
Filter convolutional layer 4	25, 50, 75, 100	25
Filter convolutional layer 5	25, 50, 75, 100	75
Dropout for all layers	0.25, 0.50, 0.75	0.50
First layer kernel size	(15, 3), (15, 6), (15, 12), (15, 18)	(15, 3)

**Table 4:** Parameters of the grid search for the repetition counting model.

Parameter	Candidates
Batch normalization	Yes, No
Normalized input	Yes, No
Activation function	relu, elu
Two extra dense layers	Yes, No
Input shape	W×18, W×3×6

**Table 5:** Comparison of 5-fold cross validation test accuracy for different combinations of sensors, with T=4 s and γ=0.95.

Inputs	5-cv Test acc
All	99.96%
Hand	95.90%
Foot	86.30%
Hand accelerometer	95.73%
Hand gyroscope	28.60%
Hand gyroscope and accelerometer	98.91%
Hand orientation	11.28%

**Table 6:** Recognized exercises in the 1-2-3 scheme workouts. P1 performed the workout with a 1-3-5 scheme.

Participant	1-rep	2-rep	3-rep	5-rep
P1	4/10	-	9/10	10/10
P3	10/10	10/10	9/10	-
P4	7/9	9/9	9/9	-
P5	7/10	10/10	10/10	-

**Table 7:** Repetition counting performance for the constrained setting. For each exercise, we indicate the mean absolute error (MAE), mean relative error (MRE), and error distribution.

Exercise	MAE	MRE	|e| = 0	|e| = 1	|e| = 2	|e| > 2
Push-up	1.22	8.5%	58.0%	28.0%	4.0%	10.0%
Pull-up	0.91	14.0%	65.1%	23.3%	0.0%	11.6%
Burpee	0.27	2.3%	85.1%	8.5%	2.1%	4.3%
Kettlebell deadlift	0.19	1.4%	87.5%	6.3%	6.3%	0.0%
Box jump	0.47	3.9%	78.7%	12.8%	0.0%	8.5%
Air squat	1.82	12.2%	54.5%	25.0%	2.3%	18.2%
Sit-up	0.02	0.2%	97.6%	2.4%	0.0%	0.0%
Wall ball	0.26	2.3%	95.2%	0.0%	0.0%	4.8%
Kettlebell press	0.61	6.6%	54.5%	38.6%	4.5%	2.3%
Kettlebell thruster	1.18	9.2%	59.0%	28.2%	2.6%	10.3%
Total	0.70	6.1%	73.5%	17.3%	2.2%	7.0%

**Table 8:** Predicted repetition number versus actual number of repetitions of the unconstrained workout for five participants. n.r.: Not recognized, -: Not performed.

Exercise	P1	P2	P3	P4	P5	MAE	MRE
Push-up	10/10	10/10	1/10	9/10	10/10	2.0	20%
Pull-up	9/10	9/10	8/10	6/7	11/10	1.2	13%
Burpee	11/10	10/10	10/10	10/10	10/10	0.2	2%
Kettlebell deadlift	9/10	10/10	10/10	10/10	12/10	0.6	6%
Box jump	8/10	10/10	9/10	-	9/10	1.0	10%
Air squat	11/10	12/10	9/10	n.r.	10/10	1.0	10%
Sit-up	11/10	10/10	10/10	10/10	10/10	0.2	2%
Wall ball	10/10	8/10	9/10	10/10	10/10	0.6	6%
Kettlebell press	10/10	9/10	10/10	10/10	8/10	0.6	6%
Kettlebell thurster	10/10	10/10	8/10	8/10	10/10	0.8	8%

**Table 9:** Predicted number of repetitions for 1, 2, 3, and 5 performed repetitions. n.r.: Not recognized, -: Not performed

	E1	E2	E3	E4	E5	E6	E7	E8	E9	E10
	1 repetition									
P1	n.r.	n.r.	2/1	n.r.	n.r.	n.r.	2/1	1/1	n.r.	1/1
P3	0/1	1/1.	2/1	2/1	1/1	0/1	2/1	2/1	0/1	1/1
P4	1/1	n.r.	1/1	n.r.	-	1/1/	1/1	1/1	1/1	1/1
P5	n.r.	0/1	1/1	2/1	1/1	n.r.	2/1	1/1	n.r.	2/1
	2 repetitions									
P3	0/1	1/2	3/2	3/2	2/2	2/2	0/2	3/2	1/2	2/2
P4	1/2	1/2	2/2	2/2	-	2/2	2/2	2/2	2/2	1/2
P5	1/2	1/2	3/2	2/2	2/2	1/2	3/2	1/2	1/2	0/2
	3 repetitions									
P1	1/3	1/3	3/3	3/3	2/3	3/3	4/3	3/3	n.r.	3/3
P3	1/3	3/3	4/3	n.r.	3/3	2/3	4/3	4/3	2/3	3/3
P4	2/3	2/3	3/3	2/3	-	2/3	3/3	2/3	3/3	1/3
P5	2/3	0/3	3/3	3/3	1/3	0/3	5/3	1/3	3/3	0/3
	5 repetitions									
P1	0/5	4/5	5/5	4/5	5/5	5/5	5/5	5/5	3/5	5/5

## Appendix A. Exercise Recognition Model Hyper-Parameters

**Table A1:** All architecture parameters for the recognition model. CL: Convolution layer, DL: Dense layer.

Parameter	Value
Input shape	500×18×1
Convolutional filters CL1	100
Kernel size CL1	(15, 3)
Strides CL1	(1, 3)
Convolutional filters CL2	25
Kernel size CL2	(15, 18)
Strides CL2	(3, 1)
Convolutional filters CL3	75
Kernel size CL3	(15, 18)
Strides CL3	(3, 1)
Convolutional filters CL4	75
Kernel size CL4	(15, 18)
Strides CL4	(3, 1)
Convolutional filters CL5	25
Kernel size CL5	(15, 18)
Strides CL5	(3, 1)
Activation function CL1, CL2, CL3, CL4, CL5	relu
Dropout CL1, CL2, CL3, CL4, CL5	0.50
DL1 neurons	250
DL2 neurons	10
Activation function DL2	softmax

## Appendix B. Repetition Counting Model Hyper-Parameters

**Table A2:** Final hyper-parameters for the repetition counting models.

Parameters	E1	E2	E3	E4	E5	E6	E7	E8	E9	E10
Convolutional layers	5	5	5	3	5	5	5	5	5	5
Input normalization	No	yes	No	No	No	Yes	No	No	No	No
Input shape	W×3×6	W×3×6	W×18	W×3×6	W×3×6	W×18	W×3×6	W×18	W×3×6	W×18
Batch normalization	No	No	No	No	No	No	No	No	Yes	No
Activation function	relu	relu	relu	relu	relu	elu	relu	elu	relu	relu
W = T×100 [samples]	120	200	250	200	200	150	200	200	150	150
Convolutional strides	(1, 1)	(3, 1)	(3, 1)	(1, 1)	(3, 1)	(3, 1)	(3, 1)	(3, 1)	(3, 1)	(3, 1)
Dropout	Yes	Yes	Yes	No	Yes	Yes	Yes	No	Yes	No

## Appendix C. Raw Sensor Signal Plots

Raw sensor readings of all 10 exercises for the wrist-worn smartwatch of one participant. The vertical lines indicate the vibration signals.
